# The effect of parental expectancy value beliefs on children's physical activity: the chain mediating role of parental exercise commitment and children's exercise self-efficacy

**DOI:** 10.3389/fpsyg.2025.1599121

**Published:** 2025-08-06

**Authors:** Wei Xu, Chaochao Hu, Enze Zhang, Chunyu Xiang, Jiapeng Yang

**Affiliations:** ^1^Wushu School, Guangzhou Sport University, Guangzhou, China; ^2^School of Sports and Health, Guangdong Institute of Science and Technology, Zhuhai, China; ^3^Lingnan Traditional Sports Center Inheritance and Development Research Center, Guangzhou Sport University, Guangzhou, China

**Keywords:** expectation value beliefs, physical activity, exercise input, self-efficacy, chain mediating role, children

## Abstract

**Objective:**

Grounded in the expectancy-value theory, this study examined how parental expectancy-value beliefs influence children's physical activity, as well as the mediating roles of parental exercise inputs and children's exercise self-efficacy, with children serving as the research subjects.

**Methods:**

Data were collected from 1,284 children and their parents in South China using the *Exercise Expectancy and Value Scale, Parental Exercise Input Quality Scale, Exercise Health Beliefs Scale*, and *Physical Activity Rating Scale*. Path analysis was conducted employing structural equation modeling techniques.

**Results:**

(1) Parental expectancy-value beliefs, parental exercise input, children's exercise self-efficacy, and children's physical activity exhibited significant positive correlations (P ≤ 0.05). (2) Parental expectancy-value beliefs had a positive direct effect on children's physical activity (β = 8.898, 95% CI [4.754, 13.041]). (3) Parental exercise input positively influenced children's exercise self-efficacy (β = 1.178, 95% CI [0.147, 2.323]), which subsequently positively affected children's physical activity (β = 3.028, 95% CI [1.630, 4.603]). Furthermore, the chain mediation of parental exercise input and children's exercise self-efficacy indirectly influenced children's physical activity (β = 0.974, 95% CI [0.564, 1.498]).

**Conclusion:**

Parental expectancy-value beliefs significantly enhanced both parental exercise input and children's exercise self-efficacy, which subsequently promoted children's physical activity behavior. Future interventions aimed at improving children's PA levels should prioritize strengthening parental expectation beliefs, enhancing parental input to exercise, and boosting children's exercise self-efficacy.

## Introduction

With the development of modern technology, the exercise behavior of children and adolescents around the world is gradually being replaced by intelligent and convenient alternatives. According to a survey, ~80% of children and adolescents worldwide fail to meet the World Health Organization's recommended standard of at least 1 h of daily physical activity (PA) (Fan and Cao, 2017). When comparing the results of surveys on the overall PA levels of children and adolescents across different countries during the same time period, it was found that the compliance rate among Chinese children and adolescents regarding the recommended amount of PA was only 8.9%. Although this rate has shown improvement in recent years (13.1%), the pace of increase still falls short of the growth rate required and the health needs of children and adolescents (Health and China, 2023). Therefore, the primary challenge in enhancing PA among children and adolescents lies in ensuring the sustainability of their participation.

Currently, the main initiatives to promote PA among children and adolescents are focused on campuses, but schools still struggle to effectively address the problem of insufficient intrinsic motivation for children and adolescents to participate in PA, and students' physical fitness has declined consecutively for many years (Kong et al., 2020). Studies have shown that the mechanisms influencing the PA behavior of children and adolescents are complex and extensive, and parents may be the most critical factor among them (Sallis et al., 2000). Imbalances in PA are often rooted in an individual's childhood family experiences and have a lasting impact. Parents remain dominant in driving children's PA development, as children are less autonomous and have yet to form stable peer social networks. Related studies have found that children's PA is a complex behavioral process, and the construction of their physical behavior is influenced by a variety of factors, with parental expectation value beliefs possibly being an important influence (Xinlan, 2006). Eccles et al. (1998) define parental expectation value beliefs as the expectations and value judgments that parents have regarding what their children should achieve in PA. Such parental expectations not only stimulate children's intrinsic motivation but also promote active participation in PA by providing the necessary support and resources (Xin et al., 2014). Therefore, it is necessary to conduct an in-depth exploration of the relationship between parental expectation value beliefs and children's PA.

Existing studies have shown that most of them focus only on the direct relationship between parents' expected value beliefs and children's PA (Lau and Leung, 2003). However, there is still a lack of effective variables at the mediator level to reveal the relationship between the two, which may be an important reason for the discrepancy in research results. Therefore, the present study attempted to introduce mediating variables to explain this discrepancy. In order to highlight the correlation between beliefs and behaviors, in conjunction with the expected value theory, the present study chose parental exercise input and children's exercise self-efficacy as mediating variables.

Parental exercise commitment refers to the extent to which parents read their children's time, energy, and material investment in exercise (Stein and Raedeke's, 1999). Children's exercise self-efficacy refers to children's beliefs about their ability to perform or accomplish a skill or exercise goal (Perry et al., 2012). Research has shown that parents can significantly influence children's PA participation through verbal encouragement, behavioral modeling, and resource provision (Guande and Wanjiao, 2024). A study by Guo et al. also confirmed that parental role modeling, direct involvement, material support, and moral support have a positive effect on PA among children and adolescents (Ru et al., 2023). Thus, it can be seen that multiple inputs such as parental accompaniment, support, and guidance collectively influence children's PA choices and development. In addition, Rutchick et al.'s (2009) study found that parental expectations not only influence children's behavioral performance but may also have a profound effect on children's self-efficacy in PA. An individual's self-efficacy influences self-selection of PA and significantly contributes to an individual's sport participation (Siyu and Tao, 2023).

However, prior research has primarily focused on examining the prediction and influence of macro- and meso-level factors (society, school, family, etc.) on children's PA, while limited research has explored factors that directly and potentially shape parental (individual) beliefs regarding their children's PA. Existing research has predominantly examined associations between family factors and PA while overlooking how various factors influence children's PA through parental mediation. Consequently, this study explores the mechanisms underlying the relationship between parents' expected value beliefs and children's PA by introducing parental exercise commitment and children's exercise self-efficacy as mediating variables, grounded in the parental socialization model of expected value theory. This approach offers a novel contribution to recent research on children's PA by examining micro-mediated mechanisms through which parents influence their children's PA participation.

### Theoretical basis

According to G. Cust, the Knowledge, Attitude/Belief, and Practice theory is a behavior change framework applied at the individual level. The theory divides the process of individual behavior formation into three distinct stages: accumulation of knowledge (basic cognition), formation of attitudes (expectancy-value beliefs), and promotion of behavior (PA). In this framework, beliefs and attitudes serve as the primary drivers of behavior change.

According to modern expectancy-value theory, individual behavioral choices are based on a comprehensive assessment of the expectation of success (expectancy) and the value of the behavioral outcome (value) of a particular behavior. The model of parental socialization put forward by Eccles points out that in the process of interacting with their parents, children's beliefs are influenced by their parents, and they learn to conform to the behavioral patterns of the established roles. Referring to the research on the relationship between parents and children, it can be found that parents' beliefs can influence children's behaviors and attitudes in various ways (Grolnick and Ryan, 1989). Parental influence on children's beliefs about sport participation and behavioral performance is likely to be transmitted through three pathways: role modeling, experience interpretation, and experience provision, i.e., parents' beliefs about their children's participation in sports may be manifested through their inputs related to their children's sports (Eccles et al., 1998). Thus, children's beliefs and behaviors are a manifestation of parents' beliefs and behaviors, and parents change children's behaviors by influencing children's beliefs with their behaviors through their own beliefs about the value of sport expectations (Xin et al., 2013).

Through an analysis of the relationship between parents' and children's beliefs and behaviors, we determined that parents' expectancy-value beliefs regarding their children's exercise predicted their input to supporting their children's exercise engagement. These input behaviors subsequently influenced children's self-efficacy beliefs about exercise. Consequently, this study developed a modified moistening transmission mechanism of parental socialization theory, grounded in the socialization model of parental expectancy-value theory, to clarify how parents' expectancy-value beliefs affect children's PA outcomes.

### Parental expected value beliefs and children's physical activity

Currently, there are inconsistent findings regarding the influence of parental expectancy value beliefs and children's PA. Dempsey, Kimiecit, and others examined the influence of parental expectancy value beliefs on adolescents' participation in moderate-to-high-intensity PA, and the results showed that parental perceptions of adolescents' athleticism were significantly positively correlated with exercise behaviors; specifically, adolescents demonstrated recurrent exercise behaviors when parents perceived that adolescents had decent athleticism (Dempsey et al., 1993). However, Yaffe and Hao's study showed that parental expectations and behaviors have a significant effect on children's PA, but this effect is not always positive and sometimes may even have a negative impact (Yaffe, 2018; Hao and Razman, 2024).

Theoretically, higher levels of parental expectancy value beliefs may provide children with substantial emotional support. These emotional values can provide positive feedback and increase children's willingness to engage in PA. Therefore, this paper proposes the research hypothesis H1: Parental expected value beliefs have a significant positive effect on children's PA.

### Mediating role of parental exercise input

Parental investment in children's sports encompasses not only time investment and material investment but also encompasses parental role modeling and behavioral encouragement. The role of parents in children's sports careers is not limited to “companion” but also serves as “supporter” and “guide” (Xu et al., 2020). Children exhibit a high degree of dependence on their parents in the process of growing up, and in their quest for attention, they will internalize their parents' words and actions into their own behaviors and form an identity (Koepke and Denissen, 2012). Therefore, parents' motivation to participate in physical activities has the capacity to effectively elicit children's imitative behavior and willingness to participate, thereby facilitating the improvement of children's PA level (Hong, 2024). Parents typically determine the level of commitment in PA in accordance with their expectations of their children, including time, energy, and emotion (Coakley, 2006). Simultaneously, parents' sports behaviors and PA participation have a direct impact on children and adolescents' PA levels, and parents' active participation in PA can set an example for children and motivate them to increase their PA (Yanli et al., 2024). From this vantage point, the present study posits hypothesis H2: Parental exercise engagement mediates the relationship between parental expectancy value beliefs affecting children's PA.

### Mediating role of children's exercise self-efficacy

Eccles' expected value theory posits that the intensity of an individual's expected value beliefs is shaped by diverse sociocultural factors. Among these factors, children's expected value beliefs are likely influenced by parental beliefs (Yaxin and Zhumin, 2007). Bhalla and Weiss demonstrated in their research that parental beliefs strongly correlate with children's beliefs and behaviors and that positive parental evaluations of and involvement in PA significantly shape children's attitudes and PA behaviors (Bhalla and Weiss, 2010). On the other hand, Eccles' modified parental socialization model emphasizes that parents' beliefs influence children's PA through multiple pathways, with children's beliefs serving as a critical mediating mechanism (Pugliese and Tinsley, 2007). When significant others hold expectations for an individual, the individual's identification with those expectations and the reciprocal processes between them can foster positive self-efficacy. Parents' subjective performance in forming expectant value beliefs about their children contributes to adolescents' perceived expectant value beliefs about their parents during behavioral processes, thereby fostering positive self-efficacy feelings. Therefore, the present study proposes Hypothesis H3: Children's exercise self-efficacy mediates the relationship between parental expected value beliefs and children's PA.

### Chained mediating role of parental exercise engagement and child self-efficacy

**Figure 1:**
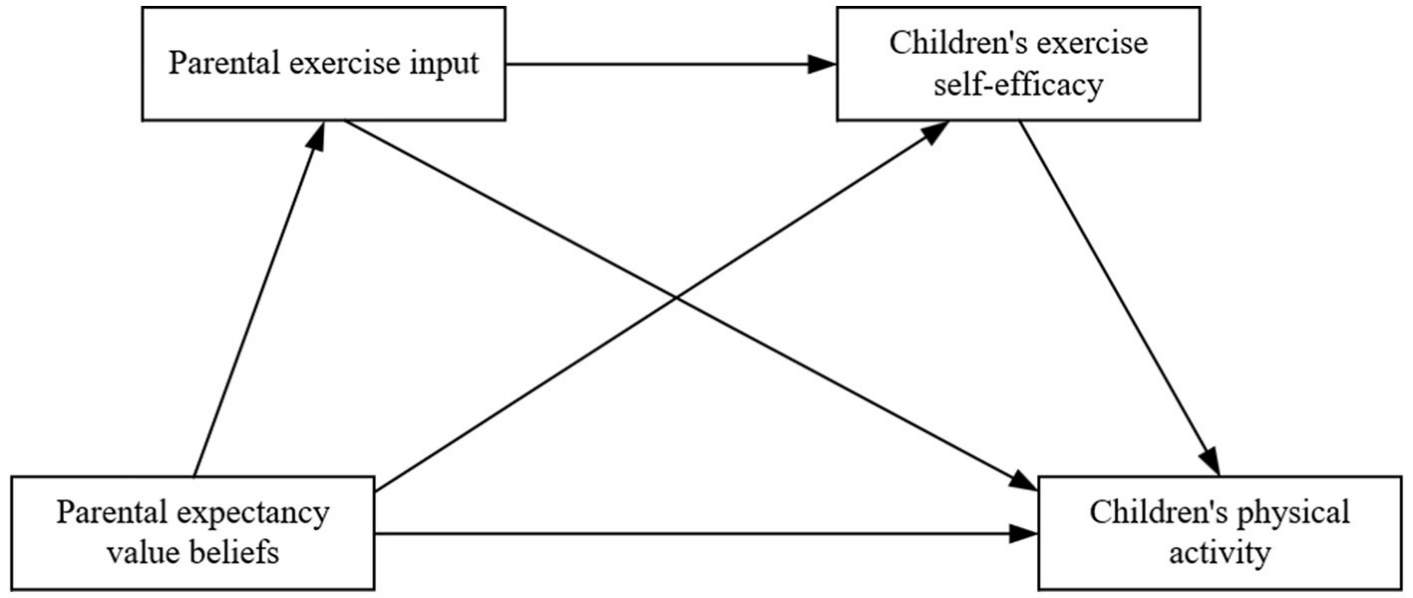
Hypothesized mediating role model.

In exploring the relationship between parental beliefs and children's PA, prior research has demonstrated that parents' expectations and value beliefs regarding their children's exercise are strongly associated with their input behaviors toward their children's PA (Wheeler et al., 2019). Furthermore, Mahoney et al. (2005) found that parental input to exercise has been identified as a crucial environmental factor enhancing children's exercise self-efficacy, which subsequently promotes their PA participation. Additionally, Brustad (1993)'s findings revealed that parental PA orientation, levels of encouragement, and children's perceived physical abilities represent significant factors influencing children's attraction to PA. Taken together, these findings suggest that parental expectancy value beliefs are manifested via parental exercise input, which further influences children's exercise self-efficacy, and these three elements collectively constitute the transmission mechanism of family members' PA effects (Figure 1). Based on these findings, the present study posits Research Hypothesis H4: Parental exercise input and children's exercise self-efficacy function as chain mediators in the relationship between parental expectancy value beliefs and children's PA.

## Materials and methods

### Participants

This study employed a mixed sampling approach, combining convenience sampling with random sampling techniques. We recruited 1,600 elementary school students and their parents from grades 4 to 6 across eight schools in South China, administering a paper-based questionnaire in an offline setting. Of the 1,600 distributed questionnaires, 316 were excluded based on established criteria for identifying invalid responses, including completion times below threshold (125 questionnaires, 7.81%), omission of critical information (94 questionnaires, 5.87%), and patterned response tendencies (97 questionnaires, 6.06%). The final analytic sample consisted of 1,284 valid questionnaires, representing an 80.25% return rate. Participants' ages ranged from 8 to 13 years (mean = 10.88 ± 0.99 years). The sample comprised 634 boys (49.4%) and 650 girls (50.6%).

### Instruments

#### Parental expectations value beliefs questionnaire

The present study used the *Exercise Expectations and Values Scale* developed by Liao and Lin (2008) to measure parents' expectancy value beliefs. The scale has 25 entries, and the original scale is divided into two subscales: children's sport expectation beliefs and value beliefs, where sport expectation beliefs are divided into two dimensions: ability expectation and demanded effort, and value beliefs are the psychological cost of failure, the expected cost of significant others, and the value of activities. The original scale was mainly developed for children's questions, so this scale was slightly modified to measure parents' sport expectation value beliefs, and a Likert 5 scale was used in order to fit the children's cognitive level. For example, (1) How good are you at sports? (2) If you were to rank the athletic ability of your classmates from worst to best, where would you rank yourself? In this study, the internal consistency (Cronbach's α coefficient) of the scale was 0.663, the KMO test value was >0.5, and the Bartlett's test of sphericity, Sig < 0.001, met the statistical requirements.

#### Parental exercise input questionnaire

This study used the *Parental Exercise Input Quality Scale* (Yaxin, 2006) developed by Lai to measure parental exercise input, which was based on Fredricks and Eccles (2008)' behavioral input entries for parents and Stein and Raedeke's (1999) conceptualization of parental input qualities and included four dimensions: parental encouragement of their sport participation, role modeling, time input, and equipment 4 dimensions. For example, (1) How much time did you and your spouse spend participating in organized sports competitions last week? (e.g., at work, in the community, etc.); (2) How much time did you and your spouse spend participating in sports with friends last week? (e.g., swimming, cycling, badminton, basketball, soccer, hiking, gymnastics, etc.). The scale questions were scored on a Likert scale, with scores assigned from 1 to 5. The original overall Cronbach's alpha coefficient was 0.85, and by combining the scores of the parents on the input entries, an overall Cronbach's alpha coefficient of 0.88 was obtained for the scale. In the present study, the Cronbach's α coefficient for the scale was 0.912, and the KMO test value was much >0.5. Bartlett's test of sphericity, Sig < 0.001, all of which met the statistical requirements.

### Children's exercise self-efficacy questionnaire

In this study, the self-efficacy dimension of the *Exercise Health Beliefs Scale* (Zhihua et al., 2006) developed by Yu was used to form the *Children's Exercise Self-Efficacy Questionnaire* to measure children's exercise self-efficacy. For example, (1) you find ways to exercise even when you are in trouble and difficulties, and (2) you find ways to exercise even when you are unhappy. The scale was scored on a Likert scale, with scores assigned from 1 to 5. In this study, the Cronbach's α coefficient of the scale was 0.843, the KMO test value was much >0.5, and the Bartlett's test of sphericity, Sig < 0.001, was in line with statistical requirements.

### Physical activity questionnaire for children

In this study, the physical activity Rating Scale (Liang, 1994) (PARS-3), revised by Liang et al., was used to measure children's PA. The scale consists of 3 items, including three dimensions of PA: intensity, duration, and frequency. The scale was assessed by the following questions: (1) What is the intensity of your physical activity? (2) How many minutes at a time do you perform physical activity at the intensity of “Question 1”? (3) How many times a month do you engage in “Topic 1” intensity physical activity? The questions are scored on a 5-point scale, with intensity and frequency of PA divided into 5 levels, from 1 to 5, and scored 1–5 respectively. The duration of PA is scored on a 5-point scale from 1 to 5, corresponding to a score of 0–4. Finally, the children's PA was calculated using the formula “exercise = intensity × time × frequency,” with higher scores indicating better PA during leisure time. The maximum score for PA is 100, and the minimum score is 0. The criteria for assessing the amount of PA are ≤19 points for a small amount of PA; 20–42 points for a medium amount of PA; and ≥43 points for a large amount of PA. In this study, the Cronbach's α coefficient of the scale was 0.604, the KMO test value was much >0.5, and the Bartlett's test of sphericity had a Sig < 0.001, which were all in line with statistical requirements.

## Results and analysis

### Common method bias test

In the statistical control phase of this study, to mitigate potential common method bias, we initially employed the Harman one-factor test to assess common method bias among the primary variables under investigation. Through principal component analysis with eigenvalue extraction set at 1, the results revealed that the variance explained by the first unrotated factor was 19.680%, falling below the 40% threshold. This finding suggests that the data in this study exhibit no substantial common method bias, thereby allowing proceeding to subsequent data analysis steps.

### Normality test

Before analyzing the data, in order to ensure that the probability density distribution of the measurement data is normal, the measurement data must be tested for normality. The normality test includes skewness and kurtosis, mainly to determine whether the measurement data have good symmetry and spikes. Kline shows that when the skewness is between ±3 and the kurtosis is between ±10, it means that the sample data of the questionnaire basically obeys the normal distribution (Kline, 2023). The distribution characteristics of the sample data were statistically analyzed with the help of SPSS software, and the results are shown in Table 1. In the normality test, the data of each variable conformed to the normal distribution, and for this reason, the study used parametric test for the subsequent analysis of each variable.

**Table 1:** Normality test table.

**Variant**	**Min**	**Max**	**Skewness**		**Kurtosis**	
			**Statistics**	**Std**.	**Statistics**	**Std**.
1 Parental expectancy value beliefs	2.12	5	0.357	0.068	2.056	0.136
2 Parental exercise input	1	5	0.612	0.068	0.943	0.136
3 Children's exercise self-efficacy	1	5	0.410	0.068	−0.469	0.136
4 Children's physical activity	0	100	0.582	0.068	−0.421	0.136

### Correlation analysis of variables

Pearson's analysis was used in this study for correlation analysis between the variables. Correlation coefficients range from −1 to 1, positive correlation coefficients range from 0 to 1, and negative correlation ranges from −1 to 0. The closer the correlation is to 1 or −1, the stronger the relationship is, and the closer the correlation is to 0, the weaker it is. The results of the correlation analysis in Table 2 show that the four variables of parental expected value beliefs, parental exercise input, children's exercise self-efficacy, and children's PA were significantly and positively correlated with each other. The correlation coefficients between parental expected value beliefs and exercise input were 0.24 (*P* < 0.001); with children's exercise self-efficacy, 0.18 (*P* < 0.001); with children's PA, 0.20 (*P* < 0.001); and with parental exercise input and children's exercise self-efficacy, 0.20 (*P* < 0.001); with children's PA, it was 0.16 (*P* < 0.001), and the correlation coefficient between children's exercise self-efficacy and children's PA was 0.36 (*P* < 0.001).

**Table 2:** Parental expected value beliefs, parental exercise input, children's exercise self-efficacy, and children's physical activity performance and correlation analysis (*N* = 1,284).

**Variant**	**M**	**SD**	**1**	**2**	**3**	**4**
1 Parental expectancy value beliefs	3.27	0.33	1	-	-	-
2 Parental exercise input	2.27	0.68	0.24^***^	1	-	-
3 Children's exercise self-efficacy	2.64	1.03	0.18^***^	0.20^***^	1	-
4 Children's physical activity	37.21	26.08	0.20^***^	0.16^***^	0.36^***^	1

* *P* < 0.05, ^**^P < 0.01,

*** *P* < 0.001. ^***^At the level of 0.001, the correlation is significant.

### Chain mediation analysis of parental exercise commitment and children's exercise self-efficacy

**Figure 2:**
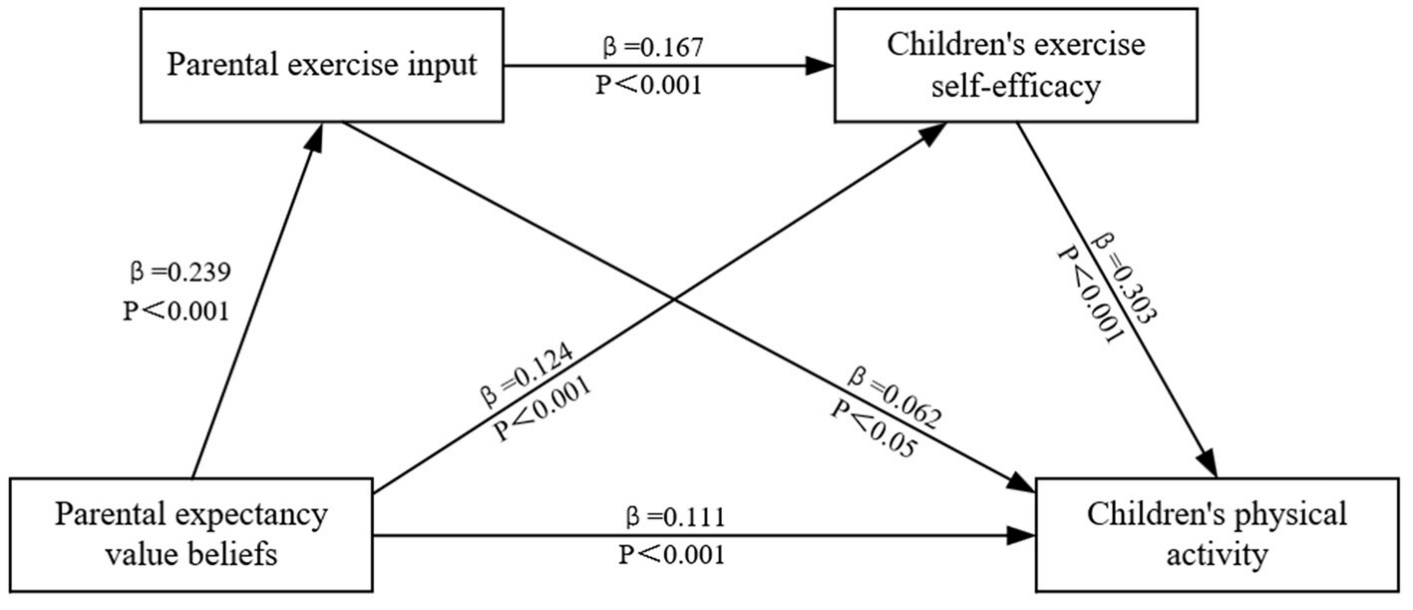
Chain mediation effect plot of parental expectation value beliefs and children's physical activity.

The regression analyses conducted in this study were performed using the nonparametric percentile Bootstrap method, model 6 of the SPSS macro program PROCESS developed by Hayes (2017) was applied to test the role of parental exercise input and children's exercise self-efficacy in the 95% confidence intervals of parental expected value beliefs about children's PA by estimating the mediating effect through 5,000 samples sampled, controlling for gender (Table 3). The chain mediating role in the predictive effect of parental exercise input and children's exercise year self-efficacy were both valid mediating variables (Figure 2). Parental expectancy-value beliefs had a positive direct effect on children's physical activity (β = 8.898, 95% CI [4.754, 13.041]). Parental exercise input positively influenced children's exercise self-efficacy (β= 1.178, 95% CI [0.147, 2.323]), which subsequently positively affected children's physical activity (β = 3.028, 95% CI [1.630, 4.603]). Furthermore, the chain mediation of parental exercise input and children's exercise self-efficacy indirectly influenced children's physical activity (β = 0.974, 95% CI [0.564, 1.498]). The results of the mediation effects test are shown in Table 4, with Bootstrap 95% confidence intervals for all indirect effects excluding 0, indicating that each mediating pathway was valid.

**Table 3:** Regression analysis of the relationship of variables in the intermediary model.

**Regression equation**		**Overall fit coefficient**			**Significance of regression coefficients**		
**Outcome variable**	**Predictor variable**	**R**	**R** ^2^	**F**	β	**S**	**t**
Parental exercise input	Genders	0.244	0.059	40.643^***^	0.028	0.037	1.034
	Parental expectancy value beliefs				0.239	0.057	8.786^***^
Children's exercise self-efficacy	Genders	0.271	0.073	33.880^***^	0.120	0.056	4.416^***^
	Parental expectancy value beliefs				0.124	0.088	4.474^***^
	Parental exercise input				0.167	0.042	6.038^***^
Children's physical activity	Genders	0.428	0.183	71.666^***^	0.176	1.337	6.881^***^
	Parental expectancy value beliefs				0.111	2.112	4.213^***^
	Parental exercise input				0.062	1.009	2.326^*^
	children's exercise self-efficacy				0.303	0.662	11.554^***^

* *P* < 0.05, ^**^P < 0.01,

*** *P* < 0.001. ^*^At a significance level of 0.05, the intermediary model is statistically significant.

**Table 4:** Effect values and 95% confidence intervals for the mediated effect pathways.

**Type of effect**	**Efficiency value**	**Bootstrap 95%CI**	
		**Lower limits**	**Upper limits**
Aggregate effect	14.078	9.858	18.298
Direct effect	8.898	4.754	13.041
Indirect effect	5.074	3.211	7.080
Parental expectancy value beliefs**→**Parental exercise input**→**Children's physical activity	1.178	0.147	2.323
Parental expectancy value beliefs**→**Children's exercise self-efficacy**→**Children's physical activity	3.028	1.630	4.603
Parental expectancy value beliefs**→**Parental exercise input**→**Children's exercise self-efficacy**→**Children's physical activity	0.974	0.564	1.498

## Discussion

### The direct effect of parental expectation value beliefs on children's physical activity

From the above analysis, it was concluded that parental expectancy value beliefs can significantly and positively influence children's and adolescents' physical activity, validating research hypothesis H1, which is consistent with previous research (Bhalla and Weiss, 2010). Parents who hold higher physical activity expectancy value beliefs usually have children who show more positive physical activity participation. Parents, as significant others in children's development, have values and belief systems that potentially shape children's behavioral patterns. When parents fully recognize the value of physical activity and have high expectations for their children's performance in this domain, they are more likely to naturally transmit this positive orientation in their lives. This reflects the unique influence of parents in the development of children's behaviors, where parental expectations serve as both an intrinsic source of motivation and an invisible guiding force that lays a solid foundation for children's physical activity behavioral trajectories.

### The simple mediating role of parental expectations value beliefs in the pathway of children's physical activity influence

Through mediating effect path analysis, this study further confirmed research hypotheses H2 and H3, indicating that both parental exercise input and children's exercise self-efficacy serve as effective mediating variables in the relationship between parental expected value beliefs and children's PA.

We found that parental expectancy value beliefs can influence children's PA by increasing parental exercise commitment and thus PA. Children were influenced by parental competence beliefs in PA and gained better exercise perceptions. Parents provide mental and behavioral support to their children through exercise input, which in turn creates a good home environment for children to grow up physically (Xinlan, 2006; Jiapeng, 2017). Parental sport inputs include providing more sport opportunities and resources, such as sport equipment, sport fields, etc., which help adolescents to participate in sport activities. Parents' expected value beliefs can significantly influence the importance they place on children's participation in PA. Parents are more likely to invest more time and resources in their adolescents' PA when they perceive PA to be of high value. For example, parents may encourage their children to participate in various sports programs, enroll their children in sports classes, or accompany their children in PA. Hong et al. (2019) showed that parents' establishment of positive sport values and increased sport expectations for their children in the family environment significantly increased children's sport participation and noted that both parents' sport attitudes and behaviors had a more significant effect on children's sport participation. If parents are actively involved in sports, their behavior will set an example for children and stimulate their interest in PA. Children are implicitly influenced to imitate their parents' exercise habits, thereby increasing their own PA.

We also found that children's exercise self-efficacy had a mediating effect on the influence of parents' expected value beliefs on children's PA. Intrinsic factors (self-efficacy, expected value) in children and adolescents affect the individual's self-perception system, which in turn influences their PA behavior (Xinlan, 2006). Parents' expectancy value beliefs do not fully determine children's PA participation but first influence children's exercise self-efficacy, which in turn influences their own PA choices and preferences. For example, even if parents place a high value on PA, children may still not be physically active if they have not developed some self-efficacy themselves. It is only when children develop the belief that “I can do it” through parental influence that they become more physically active.

### Chain mediating role of parental expectation value beliefs in the path of influence on children's physical activity

Our further analysis revealed the chain mediating role of parental exercise input and children's exercise self-efficacy in the pathway through which parental expectation value beliefs influence children's PA, thereby validating research hypothesis H4. An activity that meets with the approval of a significant other (parents) strengthens adolescents' perception of their own competence and increases their confidence levels. This enhanced perception of competence and confidence level validates the child's sense of self, thereby increasing the propensity to engage in the activity through a spiral feedback loop (Fawcett et al., 2009). Specifically, parental expectation value beliefs during PA can influence parental behaviors and perceptions of children's exercise self-efficacy, which in turn influences children's level of exercise participation. Concurrently, parental exercise engagement can influence children's exercise self-efficacy, and parental support and engagement can influence adolescents' evaluation of and confidence in their ability to perform during PA. Consequently, children's exercise self-efficacy can influence their PA participation intentions and behaviors. This chain mediation indicates an interrelated and interactive mechanism among parental expectancy value beliefs, parental exercise input, and children's exercise self-efficacy. Parental expectancy-value beliefs influenced children's PA through parental exercise input, while children's exercise self-efficacy further reinforced this influence by affecting their willingness to participate and their behavior. Concurrently, the interaction among these three variables also had a significant impact on children's PA.

## Conclusion

This study investigated the relationship between parental expected value beliefs and children's PA based on the expected value theory's model of parental socialization and explored potential mechanisms of influence. The findings suggest that parental expected value beliefs do not exclusively influence children's PA, but indirectly influence children's PA through the conversion of parental exercise inputs and the internalization of children's exercise self-efficacy. However, this mechanism of influence was not equitable due to differences in parental expected value beliefs. Parents with high expectancy-value beliefs tended to have more exercise inputs and better children's exercise self-efficacy, which in turn was more favorable to children's PA development.

## Limitations and future directions

This study explored the strong relationship between parents' expected value beliefs and children's PA, as well as the mediating roles of two variables, parents' exercise commitment and children's exercise self-efficacy, and constructed a model containing multiple mediating prediction paths through data analysis. However, there are some limitations and shortcomings. First, the selection of mediating variables and the assumptions of the variables in this study are based on the speculation of some existing research results. The influence relationship between variables may not be limited to the chained mediation paths that have been investigated in this study, and there may also be multiple chained mediation models. Therefore, subsequent studies may try to propose and test multiple path hypotheses based on different research theories and multiple variables. Second, parents' expected value beliefs and motor inputs were selected for this study rather than children's perceived expected value beliefs and motor inputs of parents. Parental beliefs and behaviors only achieve actual results through children's comprehension and understanding, and there may be corresponding differences between parents' actual beliefs and behaviors and children's perceived parental beliefs and behaviors. Finally, because the influences on PA are multifaceted, in addition to children's and parents' own subjective perceptions and behaviors, important factors such as the home physical environment and task cognition are also included. Therefore, subsequent research could explore the construction of models of influences on children's PA from multiple aspects and perspectives.

## Data Availability

The raw data supporting the conclusions of this article will be made available by the authors, without undue reservation.
